# QALY league table of Iran: a practical method for better resource allocation

**DOI:** 10.1186/s12962-020-00256-2

**Published:** 2021-01-13

**Authors:** Reza Hashempour, Behzad Raei, Majid Safaei Lari, Nasrin Abolhasanbeigi Gallezan, Ali AkbariSari

**Affiliations:** 1 Department of Health Management and Economics, School of Public Health, Tehran University of Medical Sciences, 0000-0002-2043-8451 Tehran, Iran; 2 Department of Health Economics, School of Health Management and Information Sciences, Iran University of Medical Sciences, Tehran, Iran

**Keywords:** Cost utility analysis, Economic evaluation, Cost effectiveness analysis, Iran

## Abstract

**Background:**

The limited health care resources cannot meet all the demands of the society. Thus, decision makers have to choose feasible interventions and reject the others. We aimed to collect and summarize the results of all cost utility analysis studies that were conducted in Iran and develop a Quality Adjusted Life Year (QALY) league table.

**Methods:**

A systematic mapping review was conducted to identify all cost utility analysis studies done in Iran and then map them in a table. PubMed, Embase, Cochrane library, Web of Science, as well as Iranian databases like Iran Medex, SID, Magiran, and Barakat Knowledge Network System were all searched for articles published from the inception of the databases to January 2020. Additionally, Cost per QALY or Incremental Cost Utility Ratio (ICUR) were collected from all studies. The Joanna Briggs checklist was used to assess quality appraisal.

**Results:**

In total, 51 cost-utility studies were included in the final analysis, out of which 14 studies were on cancer, six studies on coronary heart diseases. Two studies, each on hemophilia, multiple sclerosis and rheumatoid arthritis. The rest were on various other diseases. Markov model was the commonest one which has been applied to in 45% of the reviewed studies. Discount rates ranged from zero to 7.2%. The cost per QALY ranged from $ 0.144 in radiography costs for patients with some orthopedic problems to $ 4,551,521 for immune tolerance induction (ITI) therapy in hemophilia patients. High heterogeneity was revealed; therefore, it would be biased to rank interventions based on reported cost per QALY or ICUR.

**Conclusions:**

However, it is instructive and informative to collect all economic evaluation studies and summarize them in a table. The information on the table would in turn be used to redirect resources for efficient allocation. in general, it was revealed that preventive programs are cost effective interventions from different perspectives in Iran.

## Introduction

The limited healthcare resources cannot meet all the demands of the society [1] so, decision makers will have to choose feasible interventions and reject the others [2]. Thus, health systems should prioritize and use their limited resources efficiently. Economic evaluation studies is the best tool that aids priority setting and efficient resource allocation in the health sector [3, 4]. Many countries have adapted health technology assessment systems for evaluation of health interventions where in, technology and, economic evaluation, lies at the heart of any health technology assessment (HTA) [1, 3]. Cost effectiveness (CE) and cost utility (CU) are the main methods used frequently for economic evaluations in healthcare sector [4]. However, merely economic evaluation studies cannot fully guide policy-makers to a wide range of programs that might be a wise investment. To overcome this problem, cost-effectiveness threshold analysis has been developed to identify the level of cost per unit of outcome below which an intervention might be described as cost-effective [4]. In this regard, league-tables are a great instrument option for policy-makers to determine threshold values to help them make the best use of resources but, this necessitates a comprehensible league table approach in which a list of ICURs are interpreted in the context of specific costs and cost-effectiveness of competing interventions [7]. League tables rank health strategies, programs and interventions in terms of cost-effectiveness [5] for numerous diseases [6]. The intervention choices on the league table has the intervention with the lowest ICUR or cost per QALY placed at the top – and then moves down the list, to interventions with sequentially higher ratios, until the budget is used up [6–8]. They are valuable tools for prioritizing health expenses, especially for national health resources [9, 10]. It has been used as a policy tool by high [9], middle and low-income countries [5]. League tables are frequently used and they have been used for public health by WHO in the World Health Report since 2000 [11, 12]. A few regional league tables are available for some diseases. For example, there are tables for 60 different interventions in Africa [6]. The league tables are available in other countries as well [13]. Results from one of the most important studies has provided more than 3600 ICERs for more than 2000 health programs and strategies [6].

In Iran’s health system, the systematic use of economic evaluation started only few years ago but it has been expanding gradually. A league table related to public health interventions has not been developed in Iran to date. The main purpose of this paper was to assemble all cost utility studies systematically and then summarize the findings of cost utility analysis studies conducted in Iran and thereby, develop a QALY league table for the country. In doing so, decision makers would be able to distinguish and choose the best cost-effective interventions.

## Methods

In this study we aimed to gather all cost-utility studies based in Iran and summarize them in a table. PRISMA, the methodological guidance for reporting systematic reviews, was used in this study [14]. The study protocol was registered (R.H) in the international prospective register of systematic reviews database (PROSPERO). The registration number is CRD42019123313.

### Literature search

PubMed, Embase, Cochrane library, Web of Science databases as well as Iranian databases like Iran Medex, SID, Magiran, and Barakat Knowledge Network System were searched (R.H and B.R) for articles published from the start to January 2020. This review further searched the grey literature, implying documents that are often not well represented in indexing databases and usually have not been peer reviewed. National Institute for Health Research (NIHR), Google, Google Scholar and ministry of health webpage were reviewed for grey literature (R.H and B.R). We performed iterative reviews of reference lists attached to all papers selected for inclusion (R.H). The search process had no time nor language restrictions. The key words including cost utility, cost effectiveness, health technology assessment, HTA, economic evaluation, and QALY Iran were identified from our searching of respective literature on economic evaluation and health technology assessment studies, and then we conducted a search of extracted key words in aforementioned electronic bibliographic databases.

The complete search strategy in the PubMed database was as follows: cost utility [title/abstract] OR cost effectiveness[title/abstract] OR health technology assessment[title/abstract] OR HTA [title/abstract] OR economic evaluation[title/abstract] OR cost per QALY [title/abstract] AND Iran [title/abstract].

The same search strategy was adapted for other international databases using Boolean operators like OR as well as AND.

### Eligibility criteria

All cost utility studies reporting cost per QALY or ICUR and which were published till January 2020 conducted in Iran were eligible for inclusion in this review. On the contrary, all letter to editor, conference papers, review articles, cost-effectiveness, cost minimization, and cost consequences studies, and studies with low standards were excluded. Besides, all cost utility studies not done in Iran were omitted.

### Selection of articles

After removing duplicates, the titles and abstract, the papers were screened to eliminate irrelevant papers. All the steps were performed by two authors independently (R.H and B.R). Discrepancies between the reviewers were resolved by discussion or consultation with a third author (A.A). Then, the full text of the remaining papers was reviewed by the two reviewers separately (R.H and B.R) Persisting discrepancies were resolved by third author (A.A). All studies reported whether cost per QALY or ICUR in Iran were included. All low-quality studies (scored less than 6) appeared not to match our inclusion criteria and were excluded.

### Synthesis

Detailed information was extracted from each included study using a pre-structured data extraction form by two authors (M.S and N.A), separately. Any discrepancies between them were resolved through discussion; otherwise, they were resolved by the third author (R.H). Data on publication year, type of intervention (drug, screening, technology, surgery, vaccine, follow-up), year costs, sensitivity analysis (one way, two way, probabilistic sensitivity analysis and so on), perspectives adapted (society, health system, health insurance organization and so forth), discount rate, outcomes (ICUR or cost per QALY) and their recommendation were extracted. To standardize the results of studies conducted in different years, costs were deflated using the formula below:

$$Future\,value= Present\,value \times (1+r) {^n}$$

n and r are year and inflation rate respectively.

In case an article did not report the year costs, year of the paper publication was considered as a base for cost adjustment. Moreover, in case an article used Rial for calculation, we converted Rial to USD to reduce heterogeneity.

### Quality assessment

The quality of the eligible studies were determined by two independent investigators (M.S and N.A), according to the Joana Briggs Institute (JBI) quality assessment checklist [15]. The quality appraisal results were checked by a third reviewer (R.H). The JBI tool consists of the following 11 appraisal items: (1) well-defined question, (2) description of alternatives, (3) relevant costs and outcomes, (4) effectiveness, (5) outcome and cost measured accurately, (6) cost and outcome valued credibly, (7) adjusting cost and outcome for different timing, (8) incremental analysis, (9) sensitivity analysis, (10) including all concerns, and (11) generalizability. Each item was scored as 1 if the study met a criterion, and all the scores were summed up to reach a total score, which ranged from 0 (lowest quality possible) to 11 (highest quality possible). The studies were categorized into three types: the studies that scored 10 and 11 were considered as excellent quality studies (1), the studies that scored eight and nine as good quality studies (2) the studies that scored seven and six were considered as medium quality studies (3).

## Results

**Fig. 1:**
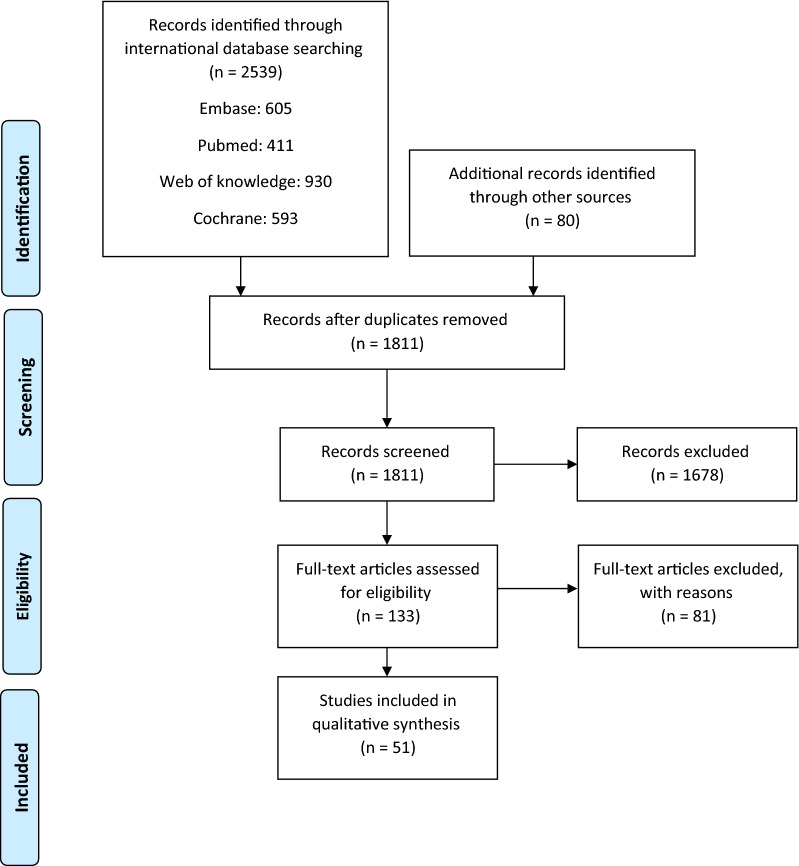
Flowchart of study and inclusion in studies

In total, our initial search yielded 2619 papers; out of which, 808 articles were duplicate and they were removed. Then, titles and abstracts of the remaining papers were reviewed, 1678 of which were excluded and 133 articles were selected. Full text of 133 articles were reviewed and 51 cost utility analysis studies were found eligible for inclusion in the final analysis (Fig. 1).

Since there was heterogeneity in the study’s results and methods, for instance, in terms of year of costing, and study perspective (viewpoint), the results of them cannot be combined or synthesized. however, QALY was the most common outcome in all included studies. Out of the 51 studies, 13 studies were based on different cancer-related interventions (e.g. screening, chemotherapy and other interventions), followed by seven studies on different programs for heart diseases, six studies were carried out on orthopedic interventions, four studies were performed on multiple sclerosis interventions, two studies were conducted on different strategies of hepatitis disease. Table 1 shows studies and diseases.

**Table 1:** The main characteristics of the studies included in the present review

Refs.	Year	Disease	Perspective	Quality appraisal
[22]	2011	Colon cancer	Health Insurance Organization	1
[25]	2019	Gastric cancer	Society	1
[18]	2014	Breast cancer	Payer	1
[20]	2008	Breast cancer	Third party	2
[71]	2012	Colorectal cancer	Health care system	1
[19]	2013	Breast cancer	Society	2
[17]	2010	Breast cancer	Health care system	1
[72]	2013	Lymphoma	Society	2
[16]	2012	Breast cancer	Health system	1
[23]	2008	Lung cancer	Health system	1
[21]	2013	Cervical cancer	Government	2
[24]	2017	Lung cancer	Health system	1
[73]	2013	Cervical cancer	Health provider	1
[30]	2000–2005	Cardiac valve dysfunction	No	3
[28]	2010	Coronary heart disease	Society	1
[74]	2013	Stroke	No	3
[29]	2015	Acute ischemic stroke	Third party payer	1
[26]	2015	Myocardial infraction	Payer	1
[27]	2014	Myocardial infraction	Payer	1
[31]	2019	venous thromboembolism prophylaxis	Payer	1
[44]	2014	Chronic Hepatitis C virus	Payer	1
[43]	2014	Chronic Hepatitis B	Society	1
[32]	2012	Orthopedic condition	Ministry of Health	1
[33]	2015	Chronic low back pain	Society	2
[33]	2018	chronic low back pain	Society	2
[34]	2014	Osteoporosis	Health system	1
[38]	2017	Osteoporosis	Health Insurance Organization	1
[36]	2019	Severe Postmenopausal Osteoporosis	Health System	1
[46]	2011	Multiple sclerosis	Health care	1
[45]	2012	Multiple sclerosis	Society	1
[47]	2019	Relapsing remitting multiple sclerosis	Society	1
	2019	Relapsing remitting multiple sclerosis	Society	1
[49]	2012	B-thalassemia	Society	1
[50]	2018	Thalassemia	Society	1
[40]	2015	Hypothyroidism	Society	2
[41]	2010	Galactosemia	Society	2
[39]	2010	Phenylketonuria	Society	2
[42]	2010	Congenital abnormalities	Society	2
[35]	2013	Refractory rheumatoid arthritis	Health service governor	1
[37]	2014	Rheumatoid arthritis	Payer	1
[53]	2014	Depression disorder	Health system	2
[45]	2014	Helicobacter pylori infection	Provider	2
[59]	2019	Dental disease	Health system	1
[57]	2019	Renal Disease	Society	1
[60]	2019	Streptococcus pharyngitis	Society	2
[55]	2019	Chronic Kidney Disease	Health Insurance Organization	1
[58]	2014	Ulcerative coitus	No	1
[56]	2019	Febrile seizure	Society	1
[52]	2016	HIV	Government	2
[61]	2018	Short stature	Health Insurance Organization	1
[51]	2011	Hemophilia a	Ministry of Health	1

Societal perspective (n = 19), health system (n = 10), and payer (n = 6) were the most common perspectives taken. Health insurance organization was considered in four studies. Provider, third party, ministry of health, and government in which each has been adapted as the viewpoint in two studies separately. Three studies did not state a perspective.

Most articles used Markov model (n = 23), followed by Decision Tree (n = 14) and two studies used both Markov and Decision Tree models. A quarter of the studies did not use any modeling (n = 12).

In 29 studies, discounting has been used. Discount rate for cost ranged from 0 to 7.2%. The discount rates were 3%, 5%, 7.2%, and 6% in thirteen, eight, four and two studies, respectively. After, performing sensitivity analysis, the results of two studies were reported with discount rates of zero, 3%, and 7.2%. and 20% in studies which used discounting for outcomes. The discount rates for outcomes were 3%, 5%, 6% and 7.2% in seventeen, six, two and four studies, respectively. Two studies used different discount rates of zero, 3% and 7/2% to perform the sensitivity analysis.

The majority of studies undertook a sensitivity analysis (n = 47). Some papers used several techniques, but Probabilistic analysis (n = 16) was the predominant technique, followed by one-way sensitivity analysis (n = 15). Sensitivity analysis was reported in two studies, but the kind of which has not been mentioned.

### Result of ICUR and cost per QALY for intervention

Multiple measures have been used for evaluating outcome data, including incremental cost, incremental QALY, ICUR, and QALY. Various methods of costing, modeling, discount rate, and perspectives had been used in the selected studies. Hence, there was high heterogeneity among variables making it difficult to rank interventions based on cost per QALY or ICUR. So, we report cost per QALY or ICUR for all interventions from different perspectives and then summarized all pivotal information in Additional file 1: Appendix S1 and Additional file 2: Appendix S2.

### Cancer

five out of twelve studies were performed on breast cancer. Mammography in the first round was cost effective in 53% of cases from the health system perspective in Iranian women aged 40–70 years based on modeling but it was not cost effective in the second and the third rounds. Cost per QALY ranged from 15.75 to 621 USD [16]. Adjuvant chemotherapy plus trastuzumab (Cost per QALY = 4,756 USD) was not a cost-effective option for treating patients with HER2-positive early breast cancer versus adjuvant chemotherapy alone (Cost per QALY = 1,115 USD) from Iranian health system perspective [17]. and intensive follow-up model was not cost-effective versus standard follow-up for breast cancer from payer perspective with cost per QALY of 178,792 USD and 381,070 USD respectively [18]. Doxorubicin and Cyclophosphamide (AC) with cost per QALY of 11,554 USD was considered as a cost-effective option for the treatment of women with advanced breast cancer who were younger than 65 years old versus Gemcitabine and Paclitaxel (PG) with cost per QALY of 16,415 USD from society perspective [19]. 5-fluorouracil, doxorubicin, cyclophosphamide (FAC) was a cost effective treatment in women less than 75 years old suffering from breast cancer with node-positive versus Docetaxel with doxorubicin and cyclophosphamide (TAC) from third-party perspective [20]. Cost per QALY for FAC and TAC were 355 USD and 5,500 USD respectively.

Quadrivalent HPV vaccine is not a cost-effective option for cervical cancer screening in girls at the age of 15 from government perspective in Iran. Moreover, cost per QALY for different strategies of cervical screening ranged from $0.5750 (no screening) to $7.866 (pap smear starting at the age of 21 and repeat every three years) from health providers perspective. It is recommended for women in Iran to start pap smear at the age of 35 and repeat it every 5 or 10 years [21].

The most cost-effective options for colorectal and colon cancer are colonoscopy screening every 10 years starting at the age of 40 and fecal immunochemical test, or colonoscopy every 10 years respectively in the target population from health care system perspective. Cost per QALY ranged from 67.3 USD (no screening) to139.1 USD (colonoscopy) [22].

Pet scan and IEV regimen (ifosfamide, epirubicin and etoposide) were cost effective alternatives in the treatment of non-small cell lung carcinoma from health system perspective and patients with lymphoma from society perspective, respectively [23].

Screening of Smokers aged 55–74 for lung cancer versus no screening is a cost-effective option from health system perspective [24].

It is suggested that oncologists use epirubicin, oxaliplatin, and capecitabine (EOX) drug regimen compared to docetaxel, cisplatin, and fluorouracil (DCF) for the treatment of patients with gastric cancer. EOX is a cost-effective drug from society’s perspective [25].

### Coronary artery disease

Aspirin is a cost-effective option in men with a 10-year CVD risk of 15% from payer perspective[ [26]] and simvastatin 10 mg is a cost-effective intervention in CVD-healthy men aged 45 with a 10-year CVD risk of 15% for the prevention of myocardial infarction from payer perspective [27].

Coronary bypass surgery (CBAG) in patients with multi-vessel coronary artery disease [28] and, tissue plasminogen activator in patients with ischemic stroke [29] are cost effective interventions from society and third-party perspective, respectively. Moreover, homograft valve in patients that underwent homograft and mechanical heart valve replacement surgery is a cost effective intervention [30].

Enoxaparin for inpatients treatment of venous thromboembolism prophylaxis with moderate to high risk is not a cost-effective option in comparison to heparin from perspective of payer in Iran [31].

### Orthopedic disease

EOS imaging technique is not cost-effective in routine practice from ministry of health perspective [32]. Electroacupuncture is more cost-effective than nonsteroidal anti-inflammatory drugs for the treatment of chronic low back pain from society perspective [33]. In another study, it is alleged that electroacupuncture is a more cost-effective intervention that NSAIDs in treatment of patients with chronic low back pain from perspective of society [33]. For osteoporosis, teriparatide is not a cost-effective intervention compared to alendronate and risedronate from health system perspective in the treatment of postmenopausal Iranian women aged 60 years and above [34]. Rituximab versus disease-modifying anti rheumatoid drugs (DMARDs) is not a cost-effective intervention for the treatment of patients with refractory rheumatoid arthritis from health service perspective [35]. Teriparatide also is a cost-effective option versus no treatment in treatment of women with Severe Postmenopausal Osteoporosis (PMO) from health system perspective [36].

Moreover, Tocilizumab plus methotrexate compared with infliximab plus methotrexate is not a cost-effective option for Rheumatoid Arthritis Patients from payer perspective [37].

Dual energy absorptiometry (DXA) & osteoporosis self- assessment tool (OST) is more cost-effective program than DXA in people over 55 years for Osteoporosis from health insurance organization perspective [38].

### Congenital disease

Screening for PKU versus no screening is beneficial to society and patients and ICUR is $33,860 [39]. The ICUR of screening versus no screening for hypothyroidism among infants is $13,413 from society perspective. Thus, the screening is not only economically beneficial, but it also, prevents mental retardation [40]. Galactosemia screening program versus no screening is both cost-effective and socially acceptable among infants and ICUR is $12,000 from society perspective [41]. ICUR of screening versus no screening for Phenylketonuria, Hypothyroidism, Galactosemia and Favism are $3386, $13,078, $19,641 and $1088 respectively from social perspective. This neonatal screening yields long term benefits [42].

### Hepatitis

In chronic hepatitis B, cost per QALY of medications ranged from $3474.78 in Tenofovir (TDF) to $10359.24 in Entecavir (ETV) in patients with HBeAg-negative chronic Hepatitis B from society perspective. Thus, TDF in patients with HBeAg-negative CHB is a highly cost-effective strategy [43].

In the treatment of patients with HCV genotype 1, the highest cost per QALY was $3826.8 for Ledipasvir and Sofosbuvir (LDV + SOF) and the least cost per QALY was $635.4 for Pegylated interferon and Ribavirin + Sofosbuvir (SOF + PR). The combination of SOF + PR was most cost-effective from payer perspective [44].

### Multiple sclerosis (MS)

For patients aged 30 years old diagnosed with relapsing multiple sclerosis, the ICUR varies from $3850 to $18,050 for different strategies. All brands of interferon beta products except Avonex is cost-effective in treatment of patients from societal perspective [45].

In another study, cost per QALY ranged from $2233.78 (symptom management) to $15529.78 (Avonex) for the treatment of patients with relapsing-remitting multiple sclerosis from the perspective of Iran’s health care perspective [46].

Moreover, alemtuzumab is a dominant intervention versus natalizumab in patients with multiple sclerosis from society perspective [47] Alemtuzumab and Natalizumab resulted in 25,475 and 28,902 dollars per QALY, respectively.

Fingolimod and natalizumab resulted in 27,368 and 7180 dollar per QALY from perspective of society in treatment of patients with multiple sclerosis [48]. it is suggested that fingolimod is used as the first priority for second-line treatment.

### Other diseases

B-thalassemia: DFX (deferasirox) is cost-effective compared to deferoxamine infusion for the treatment of iron overload in patients with b-thalassemia from the perspective of Iran’s society [49]. In another study, it was claimed that treating patients with Thalassemia major is a cost-effective intervention versus no treatment from social viewpoint [50].

Hemophilia A: low dose ITI (immune tolerance induction) is more cost-effective than other options for the treatment of hemophilia patients with inhibitors from Iranian ministry of health perspective [51].

Human immunodeficiency virus (HIV): methadone maintain treatment (MMT) is cost effective versus no MMT among iv drug users referred to the public MMT from governmental perspective [52].

Depression disorder: The repetitive transcranial magnetic stimulation is a cost-effective intervention versus electroconvulsive therapy in the treatment of depressive disorders from health system perspective [53].

Helicobacter pylori: It is recommended to avoid carbon-13 urea breath method in large scale among Iranian adult population with uninvestigated dyspepsia with no history of Non-Steroidal Anti-Inflammatory Drugs (NSAID) consumption and had no symptoms of other diseases from perspective of providers [54].

Chronic Kidney Disease(CKD): screening of CKD versus no screening in adult patient is a cost effective program from health insurance organization perspective [55].

For febrile Seizure in children, Phenobarbital and topiramate led to 1051 and 2466 dollars per QALY. Topiramate in patients with febrile seizure under five years of age is a cost-effective strategy from society perspective [56].

Renal disease: ]It is recommended that kidney transplantation is the best intervention compared to hemodialysis and peritoneal dialysis in patients with end stage renal disease from perspective of society [57].

Ulcerative coitus: conventional treatment is not a cost-effective option versus Infliximab in patients with moderate to severe ulcerative coitus [58].

Dental disease: varnish fluoride therapy versus no varnish fluoride therapy in students aged 7–12 years is a cost effective strategy from perspective of health system [59].

The best strategy in management of pharyngitis is rapid test antigen (RTA) from perspective of society. Cost per QALY ranged from $3.41 to $4.93 in diagnosis and treatment of pharyngitis [60].

Somatropin is a cost-effective option in comparison with no somatropin in treatment of children with short stature from health insurance organization perspective [61].

## Discussion

We found 51 CUA studies that were conducted between 2000 and 2020 in Iran. With regards to resource scarcity, it was apparent that the focus of economic evaluations was high on interventions for diseases that impose a growing burden on population health. Accordingly, the results of the current review highlighted that a large part of cost-utility studies concentrates on cancer which is the second major health problem in Iran. However, fewer studies (n = 6) have been undertaken on cardiovascular disease and strokes, which are responsible for roughly one-third of the mortality rates in Iran [62]. This finding shows that most of the cost-utility analysis studies have concentrated on high burden illness to allocate health resources economically.

Economic evaluation should be conducted and interpreted within clear and precise theoretical frameworks to conduct the research, and to support its interpretation [63]. The scope of the costs and benefits is determined by the selection of study perspective [64]. The predominant viewpoint in the studies analyzed was society (n = 19), which ensures addressing costs and benefits attributable to patients and society as a whole. There is a consensus among economists that the reliable perspective in economic evaluation is societal. A societal viewpoint entails that all costs and benefits should be included in the evaluation as wide as possible, irrespective of who pays or receives them [63]. It has been further observed that three studies have not stated their perspectives. Based on the review of articles, about 63% of them adopted narrow viewpoints on impeding generalizability and not including overall long-term implications in their analyses. Yet, some studies specified that perspectives failed to estimate the associated consequences concerning the adopted viewpoint.

Discounting refers to the translation of values drawn from a certain time horizon in the future to the present value that aims to make costs and benefits comparable throughout different years [65]. There is some controversy over the rate that should be employed to discount benefits and costs. Most of the countries have recommended reporting results with benefit and costs discounted at a range of 3–5 percent to ensure some consistency in the findings of economic evaluations. The current review found that majority of studies in the Iranian setting used the discount rate varied between 0 and 7.2% and 3% was the mode, which is consistent with the WHO’s guidelines on discounting [66]. Nevertheless, a few studies ignored or did not report discounting. In addition, it must be mentioned that there is no need to perform discount rate for short-run studies Since ICUR results are very sensitive to differences in discount rates, using a higher discount rate gives somewhat little weight to costs and benefits in the remote future, hence it can notably affect the decisions made.

Sensitivity analysis is performed to discover the effect of uncertainty on findings by changes in values of inputs and assumptions [67]. Researchers should perform a sensitivity analysis to assess the robustness of results. One-way sensitivity analysis was the most common technique that has been done owing to the uncertainty of a single component (e.g., by changing discount rate). However, for a more favorable validation of findings, probabilistic as well as multi-dimensional sensitivity analyses are suggested to not only assess the robustness of results but also to facilitate generalizability of findings to other settings.

From the review of articles, we found that Markov models (n = 23) were the most common analytic techniques followed by the decision tree models (n = 14). Markov models are useful when a decision problem involves a risk that is continuous over time, when the timing of events is important, and when important events may happen more than once. Representing such clinical settings with conventional decision trees is difficult and may require simplifying unrealistic assumptions. Markov models assume that a patient is always in one of the finite numbers of discrete health states, called Markov states. All events are represented as transitions from one state to another [68]. The evaluation of the studies in this review reflects a paucity of information useful for making decisions about the allocation of resources for healthcare interventions. It is actually, concerning that over 6 percent of Iranian GDP is being spent on the health system, with insufficient economic evidence.

In CUA studies, multiple domain scores from Health-Related Quality of Life (HRQoL) instruments are translated into a single summary utility score. By doing so, QALY estimates, and thus cost-utility ratios, as well as ICUR can be calculated [69]. Cost utility analyses adapt QALY measurement which is comparable and generalizable across various interventions as an instrument for comparing their value for money. Thus, ICUR is defined as the ratio of the difference in cost between two alternatives to the difference in effectiveness (QALY) between the same two alternatives. Each of CUAs included in this study has weighed the cost and effectiveness of a competing intervention against another one to give the decision-maker a precise quantitative understanding of their likely effectiveness. Based on findings in the present study, league table for Iranian CUA studies begins with $ 0.144 per-QALY ratio for radiography with the minimum ICUR in patients with any orthopedic problems from perspective of ministry of health and ends with $ 1,675,535 per QALY for immune tolerance induction (ITI) therapy with the maximum ICUR in treatment of hemophilia patients with inhibitors from perspective of health ministry. The major shortcoming of league tables for Iranian CUA studies may be the omission of much of the information that decision-makers might want to take into account when choosing alternatives. For instance, in recent years, few studies have been conducted on economic evaluation of interventions concerning cardiovascular disease while those diseases represented nearly %9 of disease burden, but 11% of the CUA studies in our review were related to them. However, there appears to be an imbalance existing between disease burden and studies.

It was revealed that screening programs related to all diseases were cost effective interventions except two studies [16, 21]. In one study [16] it was cost effective to use mammography in women aged 40–70 in 53% of trials but it was not cost effective to use it in second and third round. In the other study [21], Quadrivalent HPV vaccine was not cost effective. The reason for that may be attributed to ignoring some possible benefits. The other screening strategies were cost effective from different perspectives in Iran due to high effectiveness or low cost. So, decision makers should allocate resources to screening programs because they would use lower cost and produce more QALY. On the other hand, in treatment strategies, due to low effectiveness or high expenses, most of the options were not cost effective. In summary, we addressed the quality of CUA studies in Iran and suggest that the adherence to technical criteria needs to improve and methodological flaws in published work should be removed to ensure that economic evaluations do not mislead policy-makers and serve as tools of advocacy to witness which interventions are most cost- effectiveness (efficient). It is suggested that decision makers develop a webpage like Iranian Registry of Clinical Trials (IRCT) [70] for registration of economic evaluation studies, and generate agreed and international guidelines of what to do economic evaluations. In addition, obligating researchers to follow the determined guidelines before conducting the economic evaluation studies might increase homogeneity and comparability among the studies.

## Conclusions

League table serves as an approach which can help decision-makers to distil policy recommendations when confronting with imperfect information during the process of resource allocation in a rational way. Although economic evaluations have been conducted alongside higher heterogeneity and no ranking was performed, it is instructive and informative to collect all cost utility studies and summarize them in a table. Moreover, there was a limited number of economic evaluation studies related to different disease to make better decision for various strategies for every disease. As the findings illustrate, in general, screening programs were found cost effective interventions from different perspectives in Iran due to high effectiveness or low cost. Hence, decision makers are suggested to allocate resources to screening programs because they would use lower financial resources and produce more benefits.

### Limitations

- High heterogeneity was revealed and sorting was not carried out.
- There were a few numbers of studies to draw tables for all diseases.

## Supplementary Information

**Additional file 1: Appendix S1.** The results of technical charecteristics and cost per QALY of the studies.

**Additional file 2: Appendix S2.** The results of technical charecteristics and ICUR of the studies.

## Data Availability

All data used for this review are included in the published article.
