# Supplementary material for: QALY league table of Iran: a practical method for better resource allocation
Source: Cost Eff Resour Alloc. 2021 Jan 13;19:3. doi: 10.1186/s12962-020-00256-2 (PMC7807517; doi:10.1186/s12962-020-00256-2)
Supplement: Supplementary file 1 — Additional file 1: Appendix S1. The results of technical charecteristics and cost per QALY of the studies. [file 12962_2020_256_MOESM1_ESM.docx]

Appendix 1: The results of technical charecteristics and cost per QALY of the studies

| **Recommendation** | | | **Sensitive results?** | | **Cost per QALY (2019)** | | **Cost per QALY** | | | | **Interventions** | | | | | **Discount rate** | | | | | | **Model** | | **Perspective** | | | **Disease** | | **Year** | | **Title** | |
| --- | --- | --- | --- | --- | --- | --- | --- | --- | --- | --- | --- | --- | --- | --- | --- | --- | --- | --- | --- | --- | --- | --- | --- | --- | --- | --- | --- | --- | --- | --- | --- | --- |
|  |  |  |  |  |  |  |  |  |  |  |  |  |  |  |  | Outcome | | | Cost | | |  |  |  |  |  |  |  |  |  |  |  |
| "Annual high-sensitivity fecal occult blood testing, such as a fecal immunochemical test, or colonoscopy every 10 years offer the best value for the money in Iran". | | | Sensitive to test to detect complex adenoma | | 279  533  540  576 | | 67.3  128.8  130.4  139.1 | | | | No screening  Low-sensitivity guaiac fecal occult blood test which performed annually  Fecal immunochemical test which performed annually  Colonoscopy, performed every 10 year | | | | | 5 | | 5 | | | | Markov | | Health insurance organization | | | Colon cancer | | 2011 | | Markov's modelling for screening strategies for colorectal cancer([25](#_ENREF_25)) | |
| The intensive follow-up method is not cost-effective when compared to the standard model". | | | Cost | | 776,350  364,252 | | 381,070.5  178,792.84 | | | | Standard follow-up  Intensive model | | | | | - | | - | | | | Decision tree | | Payer | | | Breast cancer | | 2014 | | Cost-Effectiveness of Intensive Vs. Standard Follow-Up Models for Patients with Breast Cancer in Shiraz, Iran([21](#_ENREF_21)) | |
| "FAC was a dominant intervention compared to TAC in short-term. | | | Yes. Cost, utility | | 27,665  1,788 | | 5,500  355.5 | | | | TAC  FAC | | | | | - | | - | | | | N/A | | Third party | | | Breast cancer | | 2008-2010 | | Cost-Utility Analysis of Adjuvant Therapies for Breast Cancer in Iran([23](#_ENREF_23)) | |
| "Colonoscopy screening every 10 years starting at the age of 40 was the most cost-effective strategy". | | | Sensitive to cost of colonoscopy | | 168  113  120  93  82  82  75 | | 53  35.5  37.8  29.3  25.7  26  23.5 | | | | Colonoscopy screening every five years starting at age of 40 (COLO 40-5)  COLO 40- 10  COLO 50-5  COLO 50-10  COLO 50  COLO 55  No screening | | | | | 5 | | 5 | | | | Markov | | Health care system | | | Colorectal cancer | | 2012 | | Cost-effectiveness of Screening Colonoscopy in Iranian High Risk Population([75](#_ENREF_75)) | |
| It was demonstrated that AC is a cost-effective option in comparison with PG " | | | Sensitive to QALY | | 27,212  38,659 | | 11,554.5  16,415.1 | | | | AC  PG | | | | | - | | - | | | | Decision tree | | society | | | Breast cancer | | 2013 | | Cost-Utility of “Doxorubicin and Cyclophosphamide” versus “Gemcitabine and Paclitaxel” for Treatment of Patients with Breast Cancer in Iran([22](#_ENREF_22)) | |
| 12 months trastuzumab adjuvant chemotherapy in patients with early breast cancer is not a cost-effective strategy in Iran" | | | Sensitive to cost of trastuzumab, discount rate of octcome and hazard ratio | | 23,925  5,608 | | 4,756.6  1,115 | | | | Adjuvant chemotherapy plus trastuzumab  Adjuvant chemotherapy alone | | | | | 3 | | 3 | | | | Markov and Decision tree | | Health care system | | | Breast cancer | | 2010 | | Cost-Effectiveness Analysis of Trastuzumab in the AdjuvanTreatment for Early Breast Cancer([20](#_ENREF_20)) | |
| "It is recommended that oncologists use IEV instead of ESHAP in the treatment of patients with lymphoma. " | | | Sensitive to QALY | | 12,983  19,320 | | 5,512.56  8,203.33 | | | | IEV  ESHAP | | | | | - | | - | | | | Decision tree | | Society | | | Lymphoma | | 2013 | | Cost-Utility Analysis of IEV Drug Regimen Versus ESHAP Drug Regimen for the Patients With Relapsed and Refractory Hodgkin and Non-Hodgkin’s Lymphoma in Iran([76](#_ENREF_76)) | |
| "Mammography screening program was cost-effective in 53% of the cases, but incremental cost per QALY in the second and third rounds of screening are not cost effective". | | | Sensitive to recall rate of abnormal finding | | 877  50 | | 276.33  15.75  397.9  26.26  621.2  41 | | | | First round | Screening  No screening | | | | 3 | | 5 | | | | Markov and Decision tree | | Health system | | | Breast cancer | | 2012 | | Cost-Eﬀectiveness of Three Rounds of Mammography Breast Cancer Screening in Iranian Women([19](#_ENREF_19)) | |
|  |  |  |  |  | 1,262  83 | |  |  |  |  | Second round | Screening  No screening | | | |  |  |  |  |  |  |  |  |  |  |  |  |  |  |  |  |  |
|  |  |  |  |  | 1,971  130 | |  |  |  |  | Third round | Screening  No screening | | | |  |  |  |  |  |  |  |  |  |  |  |  |  |  |  |  |  |
| "The technical performance of PET scan is significantly higher than similar technologies for staging and treatment of non- small cell lung carcinoma (NSCLC). In addition, it might slightly improve the treatment process and lead to a small level of increase in the quality adjusted life year (QALY) gained by these patients making it cost-effective for the treatment of NSCLC". | | | sensitive | | 6,408  3,338  10,107  9,582  5,792  3,419  6,899  4,702  7,577  4,532  7,890  4,814  7,157  4,265 | | 1,023  533  1,613  1,530  924  546  1,101  751  1,209  724  1,259  768  1,142  681 | | | | A | Strategy 1- CT +  Strategy 1- CT -  Strategy 2- CT +  Strategy 2- CT -  Strategy 3- CT +  Strategy 3- CT -  Strategy 4- CT +  Strategy 4- CT -  Strategy 5- CT +  Strategy 5- CT -  Strategy 6- CT +  Strategy 6- CT -  Strategy 7- CT +  Strategy 7- CT - | | | | | | - | | | | Decision tree | | Health system | | | Lung cancer | | 2008 | | The Cost-Utility Analysis of PET-Scan in Diagnosis and Treatment of Non-Small Cell Lung Carcinoma in Iran([26](#_ENREF_26)) | |
|  |  |  |  |  | 7,884  4,799  10,243  9,646  6,846  4,433  7,222  5,153  8,469  5,481  8,532  5,583  8,371  5,431 | | 1,258  766  1,635  1,540  1,092  708  1,153  823  1,352  875  1,362  891  1,336  867 | | | | B | Strategy 1- CT +  Strategy 1- CT -  Strategy 2- CT +  Strategy 2- CT -  Strategy 3- CT +  Strategy 3- CT -  Strategy 4- CT +  Strategy 4- CT -  Strategy 5- CT +  Strategy 5- CT -  Strategy 6- CT +  Strategy 6- CT -  Strategy 7- CT +  Strategy 7- CT - | | | | | |  |  |  |  |  | |  |  |  |  |  |  |  |  |  |
|  |  |  |  |  | 7,552  5,331  9,724  10,162  6,455  4,705  6,768  5,346  8,002  5,719  8,039  5,772  7,849  5,636 | | 1,206  851  1,552  1,622  1,030  751  1,080  853  1,277  913  1,283  921  1,253  900 | | | | C | Strategy 1- CT +  Strategy 1- CT -  Strategy 2- CT +  Strategy 2- CT -  Strategy 3- CT +  Strategy 3- CT -  Strategy 4- CT +  Strategy 4- CT -  Strategy 5- CT +  Strategy 5- CT -  Strategy 6- CT +  Strategy 6- CT -  Strategy 7- CT +  Strategy 7- CT - | | | | | |  |  |  |  |  | |  |  |  |  |  |  |  |  |  |
|  |  |  |  |  | 11,879  8,770  9,736  10,174  8,518  6,833  8,567  7,453  9,517  7,619  9,499  7,598  10,452  8,108 | | 1,896  1,400  1,554  1,624  1,360  1,091  1,367  1,189  1,519  1,216  1,516  1,213  1,668  1,294 | | | | D | Strategy 1- CT +  Strategy 1- CT -  Strategy 2- CT +  Strategy 2- CT -  Strategy 3- CT +  Strategy 3- CT -  Strategy 4- CT +  Strategy 4- CT -  Strategy 5- CT +  Strategy 5- CT -  Strategy 6- CT +  Strategy 6- CT -  Strategy 7- CT +  Strategy 7- CT | | | | | |  |  |  |  |  | |  |  |  |  |  |  |  |  |  |
| "This study recommend organized cervical screening with HPV DNA testing for women in Iran, beginning at age 35 and repeated every 10 or 5 years". | | | Sensitive to cost of screening method and test characteristics | | 19  12  9  5  8  5  8  6  6  4  4  1 | | 7.8660  4.8867  3.8790  2.2020  3.3192  2.0566  3.2  2.7135  2.6090  1.8595  1.7571  0.5750 | | | | Pap smear 21–3  Pap smear 30–3  Pap smear 30–5  Pap smear 30–10  Pap smear 35–3  Pap smear 35–5  Pap smear 35–10  HPV 30–5  HPV 30–10  HPV 35–5  HPV 35–10  No screening | | | | | 3 | | 3 | | | | Markov | | Health provider | | | Cervical cancer | | 2013 | | Cost-Effectiveness of Different Cervical Screening Strategies in Islamic Republic of Iran: A Middle-Income Country with a Low Incidence Rate of Cervical Cancer([77](#_ENREF_77)) | |
| "Homograft valve replacement was more effective and less expensive than mechanical valve" | | | No | | 13,041  27,362 | 1,253  2,629 | | | | Homograft valve  Mechanical valve replacement surgery | | | | | No | | No | | | | N/A | | No | | | | Cardiac valve dysfunction | 2000-2005 | | Cost-effectiveness of homograft heart valve replacement surgery: an introductory study([33](#_ENREF_33)) | |  |
| "CABG is a cost-effective revascularization strategy versus PCI for patients with multivessel coronary artery disease in long-term in Iran". | | | No | | 8,078  12,860 | | 1,606  2,256.6 | | | | CBAG  PCI | | | | | 3 | | 5 | | | | Markov | | society | | | Coronary artery disease | | 2010 | | [Coronary bypass surgery versus percutaneous coronary intervention: Cost-effectiveness in Iran: A study in patients with multivessel coronary artery disease](https://www.cambridge.org/core/journals/international-journal-of-technology-assessment-in-health-care/article/coronary-bypass-surgery-versus-percutaneous-coronary-intervention-costeffectiveness-in-iran-a-study-in-patients-with-multivessel-coronary-artery-disease/527A46C419817D3570732AE93470B3E7) ([31](#_ENREF_31)) | |
| "The result revealed that home care for stroke is a good alternative". | | | Not sensitive | | 914  1,058  1,459  2,125 | | 388  449  619  902 | | | | =<39 years old  40-59  60-79  80=< | | | Hospital care | | NA | | NA | | | | N/A | | NA | | | Stroke | | 2013 | | Cost-effectiveness of home care and hospital care for stroke patients (persian)([79](#_ENREF_79)) | |
|  |  |  |  |  | 354  672  861  1,168 | | 150  285  365  496 | | | | =<39 years old  40-59  60-79  80=< | | | Home care | |  |  |  |  |  |  |  | |  |  |  |  |  |  |  |  |  |
| "Treatment with intravenous tPA is cost-effective from the perspectives of third-party payer ". | | | No | | 13,837  9,918 | | 7,444.03  5,335.68 | | | | Tissue plasminogen activator (tPA)  No Tissue plasminogen activator (tPA) | | | | | 3 | | 6 | | | | Markov | | Third party payer | | | Acute ischemic stroke | | 2015 | | Cost-effectiveness analysis of tissue plasminogen activator in acute ischemic stroke in Iran([32](#_ENREF_32)) | |
| "The use of low dose aspirin for the primary prevention of MI among Iranian men with an average risk of CVD can be considered as a highly cost-effective intervention compared to no-drug therapy" | | | Sensitive to the price of aspirin, the risk of CVD and the relative risk of being transferred from a healthy situation to non-fatal MI with Aspirin. | | 54  57  22  26 | | 29.3  30.6  11.8  14  17  17.5  5.8  6.4 | | | | Base-case  Second-scenario  Base-case  Second-scenario | | | | |  | | 3 | | | | 3 | | Semi-markov | | Payer | Myocardial infarction | | 2015 | | Cost-effectiveness evaluation of aspirin in primary prevention of myocardial infarction amongst males with average cardiovascular risk in Iran([29](#_ENREF_29)) | |
|  |  |  |  |  | 32  33  11  12 | |  |  |  |  | Base-case  Second-scenario  Base-case  Second-scenario | | | | | Treatment No treatment | | 3 | | | | 7.2 | |  |  |  |  |  |  |  |  |  |
| "The use of an OTC low dose statin (simvastatin 10 mg) for the primary  prevention of myocardial infarction (MI) in 45-year men with a 10-year CVD risk of 15 % could be considered highly  Cost-effective in Iran". | | | No | | 14  16  25  47  48  52 | | 6.7  7.8  12.3  23  23.8  25.6  3.3  3.6  .9  13.57  13.86  14.3 | | | | Base-case  Isfahan Cohort Study (ICS)  Prescription-only-medicine from 70 years  Base-case  Isfahan Cohort Study (ICS)  Prescription-only-medicine from 70 years | | | | | Treatment No treatment | | 3 | | | | 3 | | Semi-markov | | Payer | Myocardial infarction | | 2014 | | Cost-effectiveness and cost-utility analysis of OTC use of simvastatin 10 mg for the primary prevention of myocardial infarction in Iranian men ([30](#_ENREF_30)) | |
|  |  |  |  |  | 7  7  2  28  28  29 | |  |  |  |  | Base-case  Isfahan Cohort Study (ICS)  Prescription-only-medicine from 70 years  Base-case  Isfahan Cohort Study (ICS)  Prescription-only-medicine from 70 years | | | | | Treatment No treatment | | 3 | | | | 7.2 | |  |  |  |  |  |  |  |  |  |
| "The results showed that the SOF + PR drug compared with PR had a lower cost and was more effective, but compared with the LDV/SOF, in spite of its lower cost, it was less efficient. The use of SOF + PR regimen or LDV/SOF can significantly reduce the incidence of complications associated with the disease". | | | No | | 7,745  1,294  7,796 | | 3,801.5  635.4  3,826.8 | | | | PR  SOF+PR  LDV+SOF | | | | | 3 | | 7.2 | | | | Markov | | Payer | | | Chronic Hepatitis C Virus Genotype 1 Infection | | 2014 | | A Cost-Utility Analysis of Diﬀerent Antiviral Medicine Regimens in Patients With Chronic Hepatitis C Virus Genotype 1 Infection([47](#_ENREF_47)) | |
| "The use of TDF in patients with HBeAg-negative CHB seemed to be a highly cost-effective strategy". | | | No | | 12,303  11,947  13,276  21,105  7,079 | | 6,039  5,864.22  6,516.4  10,359.24  3,474.78 | | | | ADV  LAM  ADV+LAM  ETV  TDF | | | | | 3 | | 7.2 | | | | Markov simulation | | Societal | | | Chronic Hepatitis B | | 2014 | | A Cost-Utility and Cost-Eﬀectiveness Analysis of Diﬀerent Oral Antiviral Medications in Patients With HBeAg-Negative Chronic Hepatitis B in Iran: An Economic Microsimulation Decision Model  ([46](#_ENREF_46)) | |
| "EOS imaging technique cannot be considered as a cost-effective intervention in routine practice of mentioned indications" | | | Yes. Cost/ QALY | | 4  0.5 | | 1.295  0.144 | | | | EOS  CR | | Emitted radiation dose (18.8), Annual throughput | | | NA | | NA | | | | N/A | | Ministry of Health | | | Orthopedic condition | | 2012 | | EOS imaging versus current radiography: A health technology assessment study([35](#_ENREF_35)) | |
|  |  |  |  |  | 1  1 | | 0.384  0.192 | | | | EOS  CR | | Emitted radiation dose (2.15), Maximum Annual throughput | | |  |  |  |  |  |  |  |  |  |  |  |  |  |  |  |  |  |
| "The ﬁndings demonstrate that EA is more cost-effective than NSAIDs, as therefore can be considered as an alternative treatment for CLBP, with reasonable  Cost-utility". | | | No | | 1,340  1,295 | | 736  711.1 | | | | EA  NSAIDs | | | | | - | | - | | | | N/A | | social | | | Chronic low back pain | | 2015 | | Comparison of the Cost-utility Analysis of Electroacupuncture and Nonsteroidal Antiinﬂammatory Drugs in the Treatment of Chronic Low Back Pain([36](#_ENREF_36)) | |
| "Teriparatide is more expensive than risedronate and alendronate and is associated with very little increase in QALYs. A significant reduction in teriparatide price and a limit in its use only for high-risk women and for acute and short-term treatment courses can contribute to its cost-effectiveness". | | | Sensitive to cost | | 874  868  7,186 | | 429  426  3527 | | | | Risedronate  Alendronate  Teriparatide | | | | | 3 | | 3 | | | | Decision tree | | Health system | | | Osteoporosis | | 2014 | | Cost-effectiveness of teriparatide compared with alendronate and risedronate for the treatment of postmenopausal osteoporosis patients in Iran([37](#_ENREF_37)) | |
| "Conventional treatment rather than infliximab treatment is not cost-effective intervention in moderate to  severe refractory UC patients at a 5-year-timehorizon" | | | Price of drug | | 44,144  693 | | 21,668  340 | | | | Infliximab  conventional therapy | | | | | No | | 5 | | | | Markov | | no | | | Ulcerative coitus | | 2014 | | Economic Evaluation of Infliximab for Treatment of Refractory Ulcerative Colitis in Iran: Cost-Effectiveness Analysis([61](#_ENREF_61)) | |
| "The results suggest that the low-dose ITI protocol may be the most cost-effective option". | | | Sensitive to costs and drug dose | | 6,936,518  319,191  634,482  1,023,466 | | 1,675,534.8  77,101.36  153,260.84  247,221 | | | | Bon ITI  Low dose ITI  Malmo ITI  On-demand | | | | | - | | - | | | | N/A | | Ministry of health | | | Hemophilia A | | 2011 | | Cost-utility analysis of immune tolerance induction therapy versus on-demand treatment  with recombinant factor VII for hemophilia A with high titer inhibitors in Iran([54](#_ENREF_54)) | |
| "The use of each disease-modifying drugs (DMD) in patients with RRMS was associated with increased benefits compared with symptom management alone, albeit at higher costs". | | | Sensitive to time horizon, disease progression and drug cost | | 4,067  28,274  58,690  40,196  13,917 | | 2,233.78  15,529.78  32,236  22,078  7,644 | | | | Symptom management  Avonex  Betaferon  Rebif  Cinnovex | | | | | 7.2 | | 7.2 | | | | Markov | | Health care | | | Multiple sclerosis | | 2011 | | Cost-utility analysis of disease modifying drugs in relapsing-remitting multiple sclerosis in Iran([49](#_ENREF_49)) | |
| "The use of DFX instead of DFO represents a cost-effective  in patients with  B-thalassemia from Iran’s society perspective". | Sensitive to discount rate and utility | | 12,140  58,611 | | | | 3,826.9  18,475.6 | DFX  DFO | | | | | | 3 | | 5 | | | Markov | | | | | Society | | B-thalassemia | | 2012 | Cost–utility analysis of oral deferasirox versus infusional deferoxamine in transfusion-dependent b-thalassemia patients([52](#_ENREF_52)) | | |  |
| "MMT centers are cost-effective in preventing HIV infection and the access to this program should be facilitated for IDU". | No | | 6,526  0 | | | | 3,907  0 | Methadone Maintenance Treatment  No Methadone Maintenance Treatment | | | | | | - | | - | | | N/A | | | | | Government | | Human Immunodeficiency Virus | | 2016 | Cost-effectiveness of Methadone Maintenance Treatment Centers in Prevention of Human Immunodeficiency Virus([55](#_ENREF_55)) | | |  |
| It is cost-effective  To treat patients | No | | 19,702  19,586  23,257  26,791  21,501 | | | | \| 11,189/07 \| \| --- \| \| 11,123/38 \| \| 13,208/24 \| \| 15,215/34 \| \| 12,210/8 \| | Treatment  no treatment | | | | | | 3 | | 3 | | | Mrkov | | | | | Society | | Thalasemia-Major | | 2015 | Cost-utility of treatment of the patients with  Thalassemia Major in Iran | | |  |
| Smoking people aged 55-74 should be screened for lung cancer | P.S.A revealed that screening is a cost-effective program in 95% of trials | | 11.52  2.9 | | | | 7.56  1.9 | Screening  No screening | | | | | | No | | No | | | Markov | | | | | Health system | | Lung cancer | | 2017 | Cost effectiveness of lung cancer screening | | |  |
| Varnish fluoride therapy should be provided for students in primary schools | No | | 5.89  2.69 | | | | 3.86  1.77 | Varnish fluoride therapy  no Varnish fluoride therapy | | | | | | 3 | | 3 | | | Decision tree | | | | | Health system | | Dental decay | | 2017 | Cost-effectiveness analysis of varnish fluoride therapy of students in Urmia's primary schools | | |  |
| DXA&OST is the most Cost-effective intervention | No | | 589  581 | | | | 381  386.8 | DXA  DXA&OST | | | | | | no | | no | | | Decision tree and Markov | | | | | Health Insurance Organization | | Osteoporosis | | 2016 | Cost effectiveness of Osteoporosis Screening in Kerman | | |  |
| ALM is a dominant strategy for treatment of people with MS | It was revealed that ALM versus NTZ is cost effective strategy in 95.6% of trials (WTP threshold of 3GDP) | | 41,153  46,689 | | | | 25,475  28,902 | alemtuzumab  natalizumab | | | | | | 3 | | 7.2 | | | Markov | | | | | Society | | multiple sclerosis | | 2018 | Cost-effectiveness of alemtuzumab and natalizumab for relapsing-remitting multiple sclerosis treatment in Iran: decision analysis based on an indirect comparison | | |  |
| It was revealed that teriparatide is a cost-effective intervention | P.S.A revealed that Teriparatide is cost effective in 81% of trials at the threshold of 3GDP/capita | | 856  660 | | | | 713  550 | Teriparatide  Placebo | | | | | | 5 | | 7.2 | | | Markov | | | | | Health system | | Osteoporosis | | 2018 | Teriparatide in the Treatment of Severe Postmenopausal Osteoporosis: A Cost-Utility Analysis | | |  |
| "it is suggested that treatment with fingolimod be the first priority of second-line treatment for MS patients" | PSA showed that Fingolimod is cost effective in 100% of simulations for the threshold below 3GDP. | | 7,613  44,211 | | | | 7,180  27,368 | Fingolimod  Natalizumab | | | | | | 3 | | 7.2 | | | Markov | | | | | Society | | multiple sclerosis | | 2016 | Fingolimod versus natalizumab in patients with relapsing remitting multiple sclerosis: a cost-effectiveness and cost-utility study in Iran | | |  |
| Early detection of Chronic Kidney Disease is a cost-effective strategy from perspective of health insurance companies | No | | 5,110  6,687 | | | | 3,353  4,388 | screening  no screening | | | | | | 5 | | 5 | | | Markov | | | | | Health Insurance Org | | Chronic Kidney Disease | | 2017 | Cost-Effectiveness Analysis of Screening Chronic Kidney Disease in Iran | | |  |
| Topiramate is a cost-effective intervention for children with febrile seizure | No | | 3,758  1,601 | | | | 2,466  1,051 | Phenobarbital  Topiramate | | | | | | No | | No | | | Decision tree | | | | | Society | | Febrile Seizure | | 2017 | Cost-effectiveness analysis of topiramate versus phenobarbital in the treatment of children with febrile seizure in Shiraz | | |  |
| It was found that kidney transplantation is the best option compared with hemodialysis and peritoneal dialysis | PSA found that the probability of being cost effective of TX is 54.5% at the threshold of 12380$ | | 3,394  2,819  2,658 | | | | 2,227  1,850  1,744 | Hemodialysis (HD),  Peritoneal dialysis (PD)  Kidney Transplantation (TX) | | | | | | 6 | | 6 | | | Markov | | | | | Society | | Renal disease | | 2017 | Economic evaluation of End Stage Renal Disease treatments in Iran | | |  |
| It is recommended that oncologists use EOX regimen to treat gastric cancer patients. | No | | 6,004  1,205 | | | | 3,717  7,460 | EOX  DCF | | | | | | No | | No | | | N/A | | | | | Society | | Gastric Cancer | | 2016 | Cost-utility Analysis of the EOX Drug Regimen versus the DCF Drug Regimen for Patients with Advanced Gastric Cancer | | |  |
| The best strategy for management of pharyngitis is RTA alone | No | | 6  6.4  6.3  6.8  8.1  8.7 | | | | 3.41  3.64  3.57  3.89  4.63  4.93 | RTA  Treat all  Culture  Treat none  RTA then culture  RTA and culture | | | | | | no | | no | | | N/A | | | | | Society | | Pharyngitis | | 2015 | Cost and effectiveness analysis of the diagnostic and therapeutic approaches of group A Streptococcus pharyngitis management in Iran | | |  |
| Enoxaparin for VTE in patients does not seem to be a cost-effective strategy in comparison with Heparin in Iran. | 96% of iterations are both more costly and more effective. | | 3,188  3,125 | | | | 2,092  2,050 | Enoxaparin  Heparin | | | | | | No | | No | | | Decision tree | | | | | Payer | | Venous thromboembolism | | 2017 | The cost-effectiveness and cost-utility analysis of the use of enoxaparin compared with heparin for venous thromboembolism prophylaxis in medical inpatients in Iran | | |  |
| Somatropin therapy is a cost-effective intervention | No | | 123,431  505 | | | | 76,407  312 | Somatropin Therapy  No Somatropin Therapy | | | | | | 3 | | 6 | | | Decision tree | | | | | Patients | | Short stature | | 2016 | Cost-Effectiveness of Growth Hormone (Somatropin) for the Treatment of Children with Short Stature | | |  |
| The findings of this study showed that EA is more cost-effective option than NSAID | No | | 1,065  1,283 | | | | 659.26  793.9 | Electroacupuncture  NSAIDs | | | | | | No | | No | | | N/A | | | | | Society | | Chronic low back pain | | 2016 | Comparison of the cost-utility analysis of electroacupuncture and nonsteroidal antiinflammatory drugs in the treatment of chronic low back pain | | |  |
