# Supplementary material for: QALY league table of Iran: a practical method for better resource allocation
Source: Cost Eff Resour Alloc. 2021 Jan 13;19:3. doi: 10.1186/s12962-020-00256-2 (PMC7807517; doi:10.1186/s12962-020-00256-2)
Supplement: Supplementary file 2 — Additional file 2: Appendix S2. The results of technical charecteristics and ICUR of the studies. [file 12962_2020_256_MOESM2_ESM.docx]

Appendix 2: The results of technical charecteristics and ICUR of the studies

| Recommendation | Sensitive results? | ICUR (USD. 2019) | ICUR (USD) | interventions | | Discount rate | | Model | Perspective | Disease | Year | Title |
| --- | --- | --- | --- | --- | --- | --- | --- | --- | --- | --- | --- | --- |
|  |  |  |  |  |  | Outcome | Cost |  |  |  |  |  |
| "Hypothyroid screening can not only be economically beneficial, but it can also maintain the IQ of the patients, prevent mental retardation and complications in growth". | No | 24,420 | 13,413 | Screening  No screening | | - | - | Decision tree | Society | Hypothyroidism | 2015 | Cost-Utility of Screening Program for Neonatal Hypothyroidism in Iran([43](#_ENREF_43)) |
| "Screening program is both socially acceptable and cost-effective". | No | 60,359 | 12,000 | Screening  No screening | | - | - | Decision tree | Society | Galactosemia | 2010 | Neonatal Screening: Cost-utility Analysis for Galactosemia([44](#_ENREF_44)) |
| "The Phenylketonuria screening program was beneficial for both patients and society. It increased quality of life of the patients and might have long-term financial gain for the society". | Discount rate | 17,031 | 3,386 | Screening  No screening | | 3 | 3 | Decision tree | Society | Phenylketonuria | 2010 | Cost Utility of Neonatal Screening Program for Phenylketonuria in Shiraz University of Medical Sciences([42](#_ENREF_42)) |
| "The study results revealed the cost-effectiveness of execution of the neonatal screening program. The program leads to long-term beneficial outcome for the patients, financial saving for the society, and improvement of the patients’ quantity as well as quality of life". | No | 17,031  65,782  98,793  5,473 | 3,386  13,078  19,641  1,088 | Screening versus no screening for  Phenylketonuria  Hypothyroidism  Galactosemia  Favism | | 3 | 3 | Decision tree | Society | Congenital abnormalities | 2010 | Cost-Utility Analysis of Neonatal Screening Program, Shiraz University of Medical Sciences, Shiraz, Iran, 2010([45](#_ENREF_45)) |
| "Rituximab for treatment of patients with refractory rheumatoid arthritis is not a cost-effective in Iran in none of the scenarios". | No | 108,098 | 45,899.34 | Rituximab  disease-modifying antirheumatic drugs (DMARDs) | Base case | - | - | N/A | Health service governor | Refractory rheumatoid arthritis | 2013 | Rituximab as first choice for patients with refractory rheumatoid arthritis: cost‑effectiveness analysis in Iran based on a systematic review and meta‑analysis([38](#_ENREF_38)) |
|  |  | 76,274 | 32,386.89 | Rituximab  disease-modifying antirheumatic drugs (DMARDs) | Generic rituximab |  |  |  |  |  |  |  |
| "The regimen containing tocilizumab is not cost-effective as compared with an infliximab- containing regimen for patients with rheumatoid arthritis in Iran" | Price of drug | 123,867 | 60,800 | Inﬂiximab plus methotrexate  Tocilizumab plus methotrexate | | 3 | 3 | Markov | Payer | Rheumatoid Arthritis | 2014 | Cost-Effectiveness Analysis of Tocilizumab in Comparison with Inﬂiximab in Iranian Rheumatoid Arthritis Patients with Inadequate Response to tDMARDs: A Multistage Markov Model([40](#_ENREF_40)) |
| "The rTMS is more cost- effective than ECT. If the costs of rTMS decrease through providing this service by a public medical facility, the rTMS is more cost- effective than the ECT" | No | 1,450 | 711.72 | Repetitive transcranial Magnetic Stimulation  electroconvulsive | | - | - | Decision tree | Health system | Depression disorder | 2014 | Economic evaluation of resistant major depressive disorder treatment in Iranian population: a comparison between repetitive Transcranial Magnetic Stimulation with electroconvulsive([56](#_ENREF_56)) |
| "All brand INF β products are cost effective in Iran except Avonex. Results showed that a policy of encouraging accessibility to CBPs and biosimilars could make even high technology products cost-effective in low-middle middle income countries" | No | 59,871  64,620  57,260  63,776  42,769  44,048  39,169  41,532  48,035  52,191  46,976  49,688  12,772  13,784  12,213  13,603  30,305  31,209  27,755  29,426  37,760  41,026  36,929  39,058 | 18,873  20,370  18,050  20,104  13,482  13,885  12,347  13,092  15,142  16,452  14,808  15,663  4,026  4,345  3,850  4,288  9,553  9,838  8,749  9,276  11,903  12,933  11,641  12,312 | Adding (Avonex) to symptom management  Adding (Rebif) to symptom management  Adding (Betaferon) to symptom management  Adding IM (CBPs) to symptom management  Adding SC (CBPs) to symptom management  Adding (CBPs & BS) to symptom management | | 3 | 7.2 | Markov | Society | Multiple sclerosis | 2012 | Cost-effectiveness of different interferon beta products for relapsing-remitting and secondary progressive multiple sclerosis: Decision analysis based on long-term clinical data and switchable treatments([48](#_ENREF_48)) |
| "Quadrivalent HPV vaccine (Gardasil) is not cost-effective in Iran based on the base-case parameters value". | No | 41,803 | 17,749.8 | Adding vaccination of 15-year-old girls to existing situation (no official screening) | | 3 | 3 | N/A | Government | Cervical cancer | 2013 | Cost-Effectiveness Evaluation of Quadrivalent Human Papilloma Virus Vaccine for HPV-Related Disease in Iran([24](#_ENREF_24)) |
| "With respect to the existing prevalence of  Helicobacter pylori infection in Iran, owing to the high price of the carbon-13 urea  breath method, and lack of significant cost-effectiveness  and cost utility superiority over the serology and histology, we recommend avoiding its application in large scale and for large population groups" | No | 460  1,163 | 225.6  570.6 | Histology as  first diagnostic  test  Serology as  first diagnostic  test  13C-UBT as  first diagnostic  test | PPI based Triple Therapy | NA | NA | Markov | provider | Helicobacter pylori infection | 2014 | Economic evaluation of test-and-treat and empirical treatment strategies in the eradication of Helicobacter pylori infection; A Markov model in an Iranian adult population([57](#_ENREF_57)) |
|  |  | 175  184 | 85.8  90.44 | 13C-UBT as  first diagnostic  test  Histology as  first diagnostic  test  Serology as  first diagnostic  test | Antisecretory |  |  |  |  |  |  |  |
